# Effects of weight loss intervention on body composition and blood pressure among overweight and obese women: findings from the MyBFF@home study

**DOI:** 10.1186/s12905-018-0592-2

**Published:** 2018-07-19

**Authors:** Mansor Fazliana, Ahmad Zamri Liyana, Azahadi Omar, Rashidah Ambak, Noor Safiza Mohamad Nor, Ummi Kalthom Shamsudin, Narul Aida Salleh, Tahir Aris

**Affiliations:** 1 Diabetes & Endocrine Unit, Institute for Medical Research, National Institutes of Health, Ministry of Health Malaysia, Jalan Pahang, 50588 Kuala Lumpur, Malaysia; 2 Institute for Public Health, National Institutes of Health, Ministry of Health Malaysia, Kuala Lumpur, Malaysia; 3 Public Health Development Division, Ministry of Health, Putrajaya, Malaysia; 4 Kuala Lumpur Health Clinic, Jalan Temerloh, Kuala Lumpur, Malaysia

**Keywords:** Obesity, Body composition, Blood pressure, Lifestyle intervention, Bioelectrical impedance analyser

## Abstract

**Background:**

Obesity is related to the increased incidence of hypertension and in healthy individuals, blood pressure changes with age and body mass. The aims of this paper were to evaluate the effectiveness of the weight loss intervention on body composition and blood pressure, and to evaluate the relationship between these factors among housewives in the MyBFF@home study.

**Methods:**

MyBFF@home intervention was a quasi-experimental study which involved 328 overweight and obese housewives aged 18–59 years old (Control group: 159, Intervention group: 169). Data of the control and intervention group (pre and post intervention who completed the body composition and blood pressure measurements were analysed. Body compositions were measured using the Body Impedance Analyser (InBody 720) and blood pressure (Systolic and Diastolic) was taken using the blood pressure monitoring device (Omron HEM 907) at baseline, 6 month and 12 month. Data analyses (Pearson’s correlation test and ANOVA) were performed and analysed using SPSS Statistics for Windows, version 22.0.

**Results:**

Visceral fat area, fat mass and body fat percentage, were all significantly decreased in the intervention group compared to the control group after 6 month intervention (*p* < 0.05). Systolic blood pressure was reduced significantly by − 6.81 mmHg (95% CI: -9.72,-3.90; *p* < 0.01) in the intervention and by − 7.95 mmHg (95% CI: -11.69,-4.20; *p* < 0.01) in the control group after 6 month intervention. Diastolic blood pressure was significantly correlated with BMI (*r* = 0.19), waist circumference (*r* = 0.23), body fat mass (*r* = 0.22), body fat percentage (*r* = 0.18) visceral fat area (r = 0.22) and skeletal muscle mass (*r* = 0.14) with *p* < 0.05. At 12-month follow-up, no significant changes of blood pressure were detected in both groups.

**Conclusion:**

There were significant changes in the body fat and systolic blood pressure over 6 month among the participants in the intervention group compared to the control group. However, both groups were unable to sustain the positive changes in the body fats during the maintenance phase. There was a relationship between the body composition and blood pressure during the weight loss intervention and weight loss maintenance phase. Participation among obese housewives in a community-based intervention programme led to the improvements in blood pressure and body composition.

## Background

Adipose tissue has been traditionally considered a fat-storage organ but is now known to have an active role in systemic metabolism through the active secretion of adipokines or obesity hormones [1, 2]. Visceral adipose tissue (VAT), rather than subcutaneous adipose tissue has been shown to be profoundly responsible of most of the obesity-associated metabolic disorders and closely associated with a cluster of risk factors [3, 4]. Recent studies emphasise the importance of depots where fat is accumulated rather than the simple fat mass, and visceral obesity is known to be a strong risk factor for metabolic and subsequent cardiovascular disease [5–9]. A study among Korean premenopausal women clarified that visceral fat area is a major determinant of metabolic syndrome risk in premenopausal women compared to waist circumference [10]. Since the possibility of measuring VAT by imaging techniques such as computed tomography and magnetic resonance imaging is not always available, bioelectrical impedance analyser (BIA) represents a good alternative to estimate VAT. Patients with increased VAT-related cardiometabolic risk could be identified and this allows a better management of obese patients [11].

In healthy individuals, blood pressure (BP) changes with age and body mass [12]. Several studies have showed that overweight is one of the principal factors related to increased incidence of hypertension in worldwide [13, 14]. In 2015, the prevalence of raised blood pressured in females aged 18 and over was around 20% and males around 24% (Global Health Observatory data, WHO) [15]. In Malaysia, the overall prevalence of hypertension (known and undiagnosed) among adults of 18 years and above in the National Health and Morbidity Survey (NHMS) was 30.3% [16].

In a lifestyle intervention for obese women, the study showed significant changes in body composition, consisting of a smaller proportion of body fat and increased lean body mass [17]. Among adolescents, a combination of caloric restriction, exercise and behaviour change produced a greater decrease in resting systolic blood pressure and peak exercise diastolic blood pressure than did a programme of caloric restriction and behaviour change alone [18]. In a recent study, lifestyle-changing intervention by family physician-led group visits achieved significant weight loss and quality of life improvement in overweight and obese women [19]. In contrast, a group of older men was studied for independent and combined effects of weight loss and aerobic exercise on blood pressure. Combining the two interventions did not reduce blood pressure to a greater degree than the two interventions did independently. This difference in outcome might be explained by an age-dependent factor [20].

The objective of this study was to evaluate the effectiveness of lifestyle intervention on body composition and blood pressure among overweight and obese women who participated in the MyBFF@home study. We also aimed to evaluate the relationship between the body composition and blood pressure.

## Methods

This paper is part of the ‘My Body Is Fit and Fabulous at home’ (MyBFF@home) study. The design of the MyBFF@home was a quasi-experimental, which involved a pre and post intervention at the community setting. Participants were overweight and obese housewives living in low cost flats (People’s Home/Housing Project) in Federal Territory of Kuala Lumpur, Malaysia. The inclusion criteria were housewives aged 18–59 years old, overweight and obese with BMI 25.0 to 39.9 kg/m^2^. Participants who were morbidly obese (BMI > 40.0), currently on weight management programme, had limitation for physical activities (bed ridden and physical disability), had diabetes, heart disease, renal dysfunction and severe hypertension (confirmation with self-report) were excluded in the study. Screening of the housewives was conducted by the nurses and the medical assistants from 1Malaysia Clinics.

**Fig. 1:**
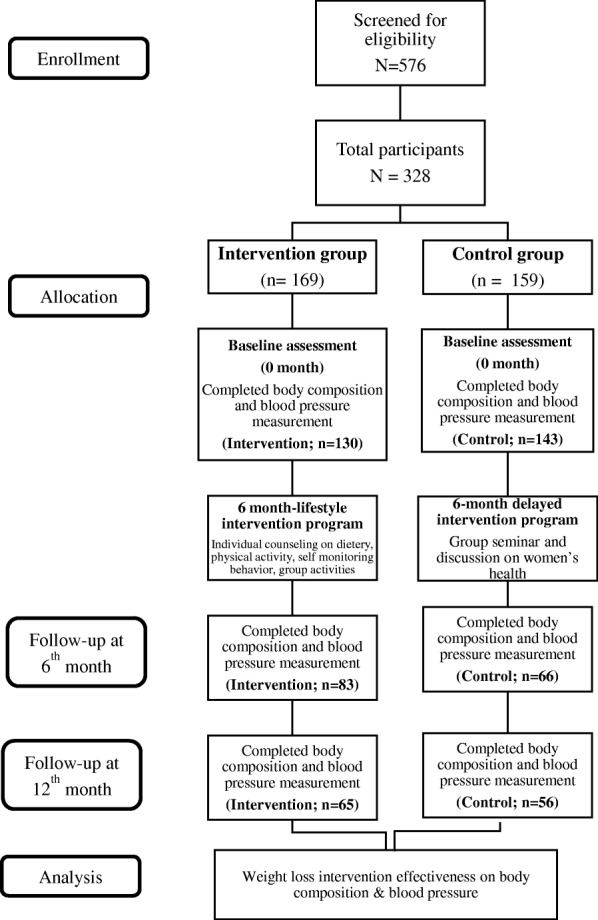
Study flow of the body composition and blood pressure data collection in the MyBFF@home study

Socio-demographic characteristics of the participants and details of the intervention were described earlier by Mohamad Nor et al. [21]. The intervention group received a weight loss intervention package consisted of individual diet counselling, group exercise and self-monitoring tools (Food diary and physical activity diary). The control group received women’s health seminar package. Both groups were followed-up for 6 months (intervention phase - intervention: *n* = 83; control: *n* = 66) and another 6 months (maintenance phase – intervention: *n* = 65; control: *n* = 56). Participants’ body mass index (BMI) and waist circumference were collected at baseline, 6 and 12 months. We only analysed participants’ data with completed body composition assessment. The study flow is represented in the Fig. 1.

### Body composition measurement

Bioelectrical impedance analyser (BIA) produces a close estimate of fat mass in a wide range of body compositions [22]. It is a non-invasive measurement of body composition and has also many advantages compared with other methods because it is inexpensive, simple, fast, safe, portable, and easy to perform, as well as requires minimum operator training [23]. Direct segmental multi-frequency BIA measurements were taken using BIA InBody 720 (BioSpace Co., Seoul, Korea). This BIA adopt a tetrapolar, eight-point tactile electrode system that separately measures impedance of the arms, trunk, and legs at six different frequencies (1, 5, 50, 250, 500, and 1000 kHz). This equipment has previously been shown to have high test-pretest reliability and accuracy [24]. Subjects had to fast for at least 5 h and not engage into strenuous physical activity during the previous 12 h.

In the MyBFF@home study, the following body composition parameters were measured: body fat mass (FM), percentage body fat (BFP), skeletal muscle mass (SMM), and visceral fat area (VFA). Testing was conducted according to manufacturer instructions. The subject stepped on the foot electrodes barefoot and stood still until body weight was measured. The subject grasped the hand electrode cables, and gently held on to the thumb electrode and the palm electrode, thereby providing contact with a total of eight electrodes. Hands were held of approximately 20 degrees away from the body, until measurements were completed. The inbuilt software was used to calculate the body composition values. The build-in body composition analysis of total weight, lean body mass and fat mass were compared to normal range values. By the end of BIA measurement, each participant was able to see her result.

### Blood pressure measurements

A fully automated blood pressure monitor using oscillometric method was used (Omron HEM 907; Omron Healthcare Europe BV, Hoofdorp, The Netherlands). Blood pressure (BP) was recorded in mmHg from the patients’ arm, in sitting position. Measurements were taken twice and a mean value was computed. Hypertension was defined by a systolic BP ≥ 140 mmHg and/or, diastolic BP ≥ 90 mmHg. Prior to recruitment, screening has been done. Therefore, those detected with moderate and severe hypertension have been referred to clinics and excluded from the study.

### Anthropometric measurements

Measurements of weight, height and waist circumference were obtained for anthropometric analysis. All participants were weighed to the nearest 0.1 kg in light clothing, without shoes and with an empty bladder on an electronic scale (Tanita HD319, Japan; at baseline, 6 month and again at 12 months). Height was measured using a SECA Bodymeter. Waist circumference was measured to the nearest 0.5 cm at the umbilicus with a flexible tape applied directly on the skin. BMI was calculated by dividing the measured body weight (kg) by the squared of body height (m^**2**^). BMI categories were referred to WHO 1998 classification.

### Statistical analysis

Baseline characteristics between study groups were compared using independent t-test. Pearson’s correlations test was done for correlation of BIA measurements with SBP and DBP during baseline. Within group changes were analysed using paired t-test. Whilst, one-way ANOVA was used to detect mean difference of body composition changes between study groups. Analyses were performed by study phase (intervention and maintenance phase). Data were analysed using IBM SPSS Statistics for Windows, version 22 (IBM Corp., Armonk, N.Y., USA).

## Results

At the baseline, the total number of participants who completed body composition and blood pressure measurement was 273 (72.2%). A total of 149 participants completed the 6-month intervention (intervention: *n* = 83; control: *n* = 66). Finally, after the next 6 months of maintenance phase, 121 participants provided all outcome data at all time points; baseline, 6- and 12-month (intervention: *n* = 65; control: *n* = 56) (Fig. 1).

Table 1 shows the baseline characteristics of body composition and blood pressure in the intervention and control group. Variables were presented as mean and standard deviations as they were normally distributed. The baseline characteristics of the measured parameters are comparable between the study and control groups. Skeletal muscle mass in the intervention group recorded higher measurement compared to control (22.35 ± 2.8 kg vs 21.67 ± 2.85 kg, *p* = 0.05). Systolic and diastolic BP were within normal range.

**Table 1:** Baseline characteristics of body composition in control and intervention group

Variables	Control			Intervention			p^a^
	n	Mean	SD	n	Mean	SD	
Age (years)	143	40.99	8.32	130	42.05	8.24	0.29
BMI (kg/m^2^)	143	31.03	4.14	130	31.48	4.14	0.37
WC (cm)	143	93.17	9.49	130	94.83	10.24	0.17
Body fat mass (kg)	143	33.11	7.67	130	34.72	8.14	0.10
Body fat percentage (%)	143	44.82	4.74	130	45.12	4.89	0.60
Visceral fat area (cm)	143	121.60	23.37	130	127.10	24.48	0.06
Skeletal muscle mass (kg)	143	21.67	2.85	130	22.35	2.80	0.05^*^
Systolic blood pressure(mmHg)	143	119.35	15.01	130	122.10	16.81	0.15
Diastolic blood pressure (mmHg)	143	77.72	10.20	130	78.40	11.32	0.60

^a^Independent t-test

^*^p < 0.01

Table 2 shows correlation between blood pressure and anthropometric and body composition indices. Diastolic BP was significantly correlated with BMI (*r* = 0.21, *p* < 0.001), waist circumference (*r* = 0.24, p < 0.001), fat mass (*r* = 0.22, p < 0.001), body fat percentage (r = 0.22, p < 0.001), visceral fat area (r = 0.22, p < 0.001) and skeletal muscle mass (*r* = 0.12, *p* < 0.05). No correlations were found between systolic BP and other parameters.

**Table 2:** The correlation between blood pressure and body composition parameters

	Systolic Blood Pressure (SP) *n* = 273		Diastolic Blood Pressure (DP) *n* = 273	
Variables	r	*p*-value	r	p-value
BMI (kg/m^2^)	0.06	0.313	0.19	0.001^*^
WC (cm)	0.09	0.131	0.23	0.000^**^
Body fat mass (kg)	0.06	0.298	0.22	0.000^**^
Body fat percentage (kg)	0.06	0.367	0.18	0.003^*^
Visceral fat area (cm^2^)	0.07	0.239	0.22	0.000^**^
Skeletal muscle mass (kg)	0.04	0.497	0.14	0.021^*^

P-value: ^*^ p < 0.01, ^**^p < 0.001

**Fig. 2:**
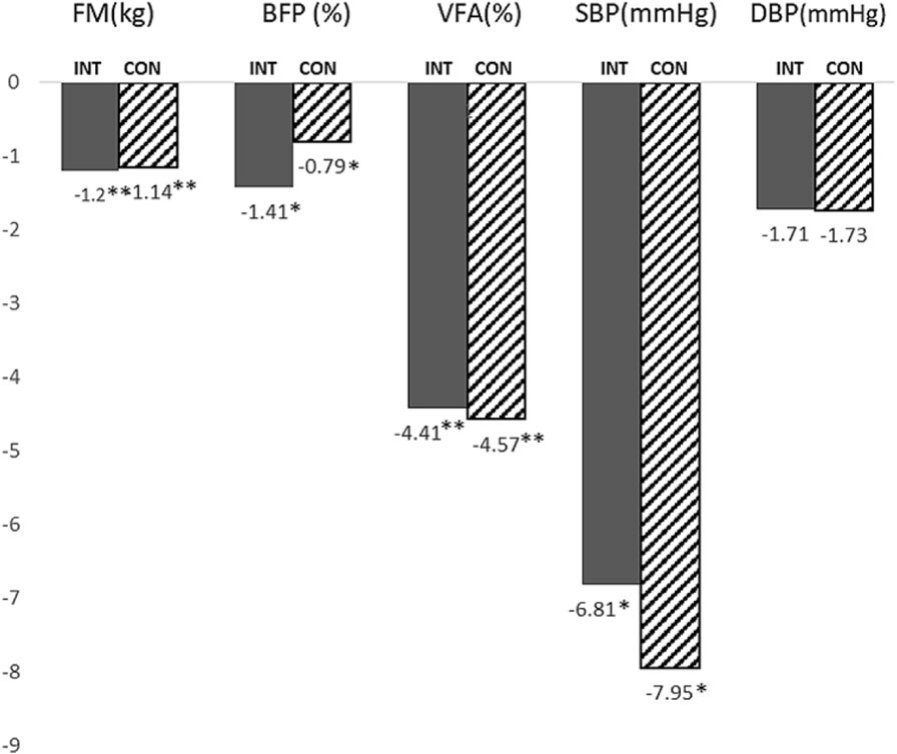
Graphical representation of changes of selected parameters at 6-month

Table 3 summarizes the body composition and blood pressure changes over the 6-month intervention period and another 6 months weight loss maintenance phase. The body composition analysis revealed a highly significant reduction of body fat mass in the intervention group, (− 1.20 kg (95% CI:-1.76,-0.64, *p* < 0.01), and also in the control group (0.48 kg (95% CI: 0.07, 0.89, *p* < 0.05). For visceral fat area, highly significant difference between baseline and month-6 were detected for intervention group (− 4.41 kg (95% CI: -6.06,-2.76, *p* < 0.001), and − 4.57 kg (95% CI:-6.83,-2.31, *p* < 0.001) for the control group. Overall, fat mass, body fat percentage and visceral fat area were reduced in both study groups, without significant difference between them. In both groups, systolic BP reduced significantly during intervention phase (p < 0.001**). Graphical changes are shown in Fig. 2.

**Table 3:** Body composition and blood pressure changes between baseline and after 6 months intervention, and between 6 month and 12 months

Intervention Phase (Baseline to 6-month)				Maintenance Phase (6-month to 12-month)		
Outcome measures	INTERVENTION (n = 83)	CONTROL (n = 66)	Between group difference ^b^	INTERVENTION (*n* = 65)	CONTROL (n = 56)	Between group difference ^b^
	Changes within group ^a^			Changes within group ^a^		
	Mean difference (95% CI)	Mean difference (95% CI)		Mean difference (95% CI)	Mean difference (95% CI)	
Body fat mass (kg)	−1.20	−1.14	0.892	0.48	0.59	0.764
	(−1.76, −0.64)	(− 1.68, − 0.60)		(0.07, 0.89)	(− 0.02, 1.19)	
	< 0.001^**^	< 0.001^**^		0.022	0.058	
Body fat percentage (%)	−1.41	−0.79	0.226	0.53	0.35	0.751
	(−2.25, −0.57)	(−1.22, − 0.36)		(− 0.41, 1.47)	(− 0.19, 0.88)	
	0.001^*^	0.001^*^		0.267	0.196	
Skeletal muscle mass (kg)	0.05	−0.14	0.202	0.32	0.00	0.016^*^
	(−0.18, 0.28)	(−0.30, 0.02)		(0.17, 0.47)	(−0.23, 0.23)	
	0.646	0.096		< 0.001^**^	0.988	
Visceral fat area (cm^2^)	− 4.41	− 4.57	0.909	2.01	1.56	0.712
	(−6.06, −2.76)	(−6.82, −2.31)		(0.51, 3.51)	(−0.44, 3.55)	
	< 0.001^**^	< 0.001^**^		0.010^*^	0.123	
Systolic BP (mmHg)	−6.81	−7.95	0.629	−1.55	−1.60	0.751
	(−9.72, −3.90)	(−11.69, −4.20)		(−4.22, 1.13)	(−5.12, 1.93)	
	< 0.001^**^	< 0.001^**^		0.252	0.368	
Diastolic BP (mmHg)	−1.71	−1.73	0.993	−1.52	−1.24	0.981
	(−3.71, 0.28)	(−4.12, 0.67)		(−3.49, 0.46)	(− 4.05, 1.56)	
	0.092	0.155		0.129	0.379	

Mean differences within group are in mean (95% CI), a negative change indicates a fall in average from baseline to 6 months

*P*-value: ^*^p < 0.01, ^**^p < 0.001

^a^Changes of body composition variables during intervention or maintenance phase analysed using paired t- test

^b^Difference of changes of body composition between intervention and control group measured using ANOVA

**Fig. 3:**
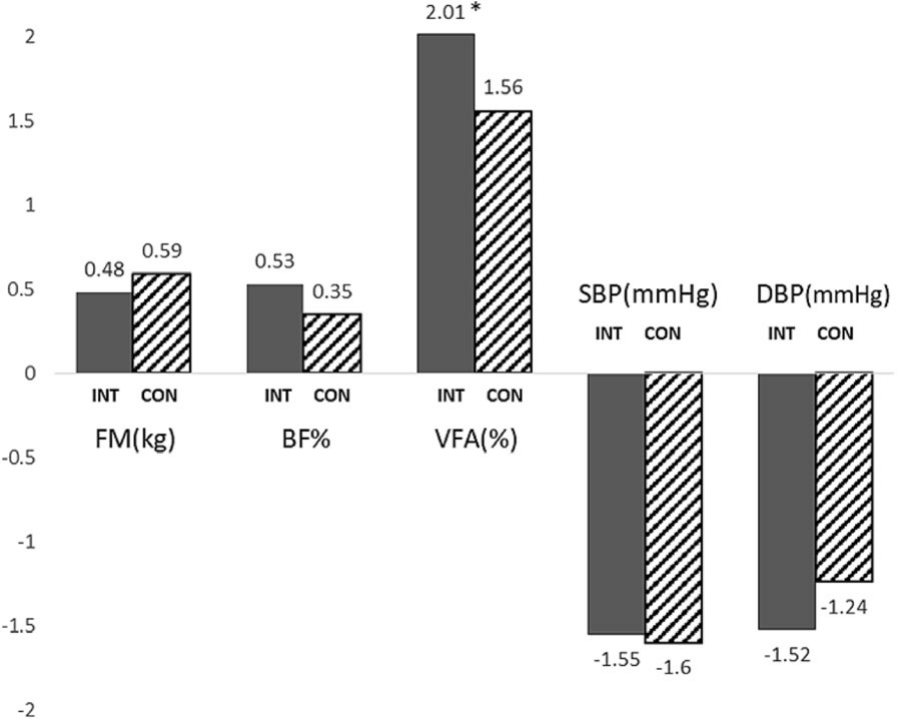
Graphical representation of changes of selected parameters at12-month

Improvement in skeletal muscle mass was retained during this phase with significant increment in the intervention compared to the control group (0.31 kg vs. 0.00 kg respectively, *p* = 0.016). Both groups gained fat content from 6 to 12 months and fat regain was significantly (*P* < 0.05) greater for the intervention than for the control group. At 12-month follow-up, systolic and diastolic BP have shown insignificant decrement in both groups (Fig. 3).

## Discussion

Our intervention programme reduced systolic blood pressure significantly, in both groups. This could be explained by reduction of fat mass, body fat percentage and visceral fat area, in both groups too. This observation was seen in other study, where in a weight loss intervention among Latinos, weight loss was followed by reduction of systolic and diastolic blood pressure [25]. However this positive effect was not only achieved among participants of the intervention arm, but also by control group participants. There is robust evidence that action to lower blood pressure does reduce the risk to health. A systematic review found that, in the populations studied, every 10 mmHg reduction in blood pressure resulted in a 17% reduction for coronary heart disease, a 27% reduction for stroke, a 28% reduction for heart failure and a significant 13% reduction in all-cause mortality [26]. In this study, systolic blood pressure was reduced significantly by 6.8 mmHg and 9 mmHg in the intervention and control group, respectively. Blood pressure showed significant positive correlations with weight, body fat, fat weight, core fat, body mass index, and basal metabolic rate in Asian obese and normal weight middle-aged women [27, 28]. This study found that subjects with higher diastolic blood pressure have higher body composition measurements. This is in line with a Chinese study by Wu O., et al. (2018) where diastolic blood pressure was significantly positively correlated with BMI and waist circumference [28]. As risk factors of the new onset of hypertension, the impact of diastolic blood pressure compared with systolic blood pressure, men compared with women, and higher body mass index were greater in the younger adults, whereas in the older adults, the impact of systolic blood pressure and female sex were greater [29].

BMI is as an important obesity index. However, the negative effect of obesity is increasingly attributed to excess adiposity, particularly central and visceral adiposity, and BMI cannot differentiate between the fat mass and other body compositions such as lean mass or bone mass. Visceral fat is an important component and has been shown to be associated with a greater cardiometabolic risk, as compared with subcutaneous fat and other obesity measurements, including BMI [30–32]. Although visceral fat area was reduced significantly at month-6 for both groups, this reduction was not maintained at 12 months. During the same period, both systolic and diastolic BP did not increase after the maintenance phase, despite increment of adiposity at 12-month. A modest weight loss can normalise blood pressure levels in hypertensive patients even without reaching ideal weight [33].

Various components in the intervention package might contribute to these findings, including the individualised dietary counselling. Recently, a dietary intervention provides significant benefits to overweight/obese patients with primary hypertension, by reduction of blood pressure, body compositions and biochemical parameters [34]. Another study showed that, a four-week diet regimen, exercise and psychological intervention for weight loss, systolic and diastolic BP showed significant decline, while the BIA analysis revealed significant reduction of body fat content [35]. One possible mechanism is through reduction of sodium intake. A Cochrane study showed that sodium reduction resulted in a decrease in SBP/DBP of 1/0 mmHg in white participants with normotension and a decrease in SBP/DBP of 5.5/2.9 mmHg in white participants with hypertension [36].

High percentage of fat, mainly visceral, have been related to insulin resistance, high levels of angiotensin II, increased secretion of aldosterone, and, consequently, high absorption of sodium in renal tubules [33, 37]. Additionally, stiffness of endothelial and vascular smooth muscle cells, extracellular matrix remodelling and perivascular adipose tissue inflammation and immune cell dysfunction contribute to the development of arterial stiffness in obesity. This is supported by Lefferts WK et al., (2017) who suggested visceral adiposity may detrimentally affect subclinical markers of cardiovascular disease risk and contribute to artery stiffness [38].

Body impedance analysis in this study revealed that skeletal muscle mass increased significantly in the intervention group at month-12. Engagement of exercise and physical activities by the intervention participants may contribute to this result [39]. This observation was not detected in the intervention period. This possibly due to a long-term effect of exercise and physical activities.

Despite participants in the control group only attended group seminars and discussion on women’s health, without the intervention package, we also saw positive effect on body composition in this group. One of the components received by both study groups was diet intake self-monitoring, where the participants needed to record their food intake routinely for at least three days in a week. Burke (2011) showed a significant association between self-monitoring and weight loss was consistently found [40]. MyBFF@home weight loss package alone might be effective, but enrolling into any weight-loss programmes might provide awareness and self-empowerment among women in the control group although they were not intervened. Control group participants may also have been previously exposed to various health programmes, conducted by the government or local communities, which may have raised health awareness among them. The participants might practice ‘Self-directed intervention’ which did not require professional assistant. Self-directed interventions are likely to be most effective when they empower participants to control and regulate their own thoughts, feelings, and behaviours, thereby changing psychological and environmental prompts to weight-gain behaviours [41]. Another example is, enrolling in a programme through work (with a corporate partner) affects retention and weight loss [42]. Control individuals in the control group who participated in the programme may be more motivated, which resulted in positive effects found in this study. Furthermore, adhering to improved lifestyle, focusing on behavioural change and maintaining weight loss after the end of the intervention seem to be the key not only for cardio-metabolic risk factors but also for sustainable health-related quality of life [43].

There are also some limitations to be mentioned. Participants were not randomly assigned to the programmes, only the location of the flats were randomly assigned. Menopausal women showed more visceral fat [44], however we do not have this data. Manufacturers of BIA technology recommend to avoid testing women when they perceive to be retaining water during the menstrual cycle. We also do not have menstrual status of the participants. However, according to a recent study, contact-electrode BIA devices can be used at any time during a woman’s menstrual cycle without altering the body composition values [45]. Another limitation is the small samples size in the subgroup, potentially influencing the power of the analyses.

## Conclusions

Participating in a lifestyle intervention programme in a community setting led to the improvements in blood pressure and the body composition. We noted significantly lower adiposity and systolic blood pressure over 6 months in the participants enrolled in the intervention programme, both in the intervention and control group. Generally, both groups were unable to show effective sustainability of positive changes in body fats during the maintenance phase. More effective strategies are needed to empower individuals to positively act on the matter of their personal health.

## Abbreviations

BFP: Percentage body fat
BIA: Bioelectrical impedance analyser
BMI: Body mass index
BP: Blood pressure
FM: Body fat mass
MyBFF@home: My Body Is Fit and Fabulous at Home
SMM: Skeletal muscle mass
VAT: Visceral adipose tissue
VFA: Visceral fat area
